# Storage Temperature Impacts on Anthocyanins Degradation, Color Changes and Haze Development in Juice of “Merlot” and “Ruby” Grapes (*Vitis vinifera*)

**DOI:** 10.3389/fnut.2018.00100

**Published:** 2018-10-25

**Authors:** Bizuayehu M. Muche, R. Alex Speers, H. P. Vasantha Rupasinghe

**Affiliations:** ^1^Department of Plant, Food, and Environmental Sciences, Faculty of Agriculture, Dalhousie University, Truro, NS, Canada; ^2^Faculty of Engineering, Canadian Institute of Fermentation Technology, Dalhousie University, Halifax, NS, Canada; ^3^International Centre of Brewing and Distilling, Heriot-Watt University, Edinburgh, Scotland

**Keywords:** storage temperature, anthocyanin, haze, polymeric color, browning, bioactives

## Abstract

This study evaluated the degradation kinetics of selected anthocyanins and the change in polymeric color, browning index, and haze development of grape juices from “Merlot” and “Ruby” grape cultivars stored at 5, 25, and 35°C for up to 360 days. Five major anthocyanins namely malvidin-3-*O*-glucoside (M3G), delphinidin-3-*O*-glucoside (D3G), petunidin-3-*O*-glucoside (Pt3G), peonidin-3-*O*-glucoside (Pn3G), and cyanidin-3-*O*-glucoside (C3G) were identified. Juice from “Merlot” had significantly higher (*p* < 0.05) content of all individual anthocyanins as compared to “Ruby.” During the long-term storage, total, and individual anthocyanins from both cultivars degraded following first-order reaction kinetics at the rate strongly dependent on temperature. At the end of the storage, noticeably higher loss of anthocyanins (95–99.9%) was observed at 25 and 35°C as compared to storage at 5°C [50–60% (“Merlot”); 74–81% (“Ruby”)]. Considerably lower rate of decay was observed at 5°C (*k* = 0.01–0.04) as compared to 25 (*k* = 0.04–0.14) and 35°C (*k* = 0.05–0.14) storage temperatures. The most temperature sensitive anthocyanin compounds were C3G (E_a_ = 66.5 kJ/mol) and D3G (E_a_ = 63.3 kJ/mol). At higher storage temperatures, significant (*p* < 0.05) and strong negative correlations were observed between anthocyanin concentrations and the levels of haze, polymeric and brown color development during storage. Storing grape juice, at lower temperature conditions could reduce the continuous loss of biologically active anthocyanins as well as the development of haze and brown color.

## Introduction

Grape juice is very important in the international juice trade because it is used not only as a single beverage but also as an ingredient for blending or making other products such as juice beverages, jams, jellies, pie fillings, and some confectionaries (1). Worldwide, the consumption of grape juice has increased from 1.07 kg per person in 1970 to 2.1 kg in 2011 (1).

Color is one of the most crucial quality parameters of grape juice and its derivatives (2). A typical purple-red color is associated with a high-quality grape juice; however change in color and the development of undesirable haze and turbidity during storage results in loss of quality and limitation of shelf life (2). The color of the grape juice is primarily due to anthocyanin pigments (3), which are responsible for the attractive color, sensory attributes and health benefits (4). Consumer awareness of dietary anthocyanins has increased in recent years because of their possible health benefits, including reducing the risk of coronary heart disease (5), stroke (6), cancer (7), and inflammation (8). However, anthocyanins are highly unstable and susceptible to various degradation reactions such as enzymatic or non-enzymatic browning (9), polymerisation (10, 11) and condensation with tannins (12) during processing and storage (13). Anthocyanin color stability depends on different factors such as pH, temperature, storage time, light, oxygen, concentration, ascorbic acid, copigments, enzymes, flavonoids, proteins, metal ions, and sugar (13–20).

In previous studies, anthocyanin degradation in different juice products was commonly evaluated as total monomeric anthocyanin, and little information is available on the fate of individual anthocyanin compounds in grape juice after long-term storage at different temperatures. Moreover, there is very limited information, to allow evaluation of the relation between anthocyanin degradation and the change in haze accumulation, polymeric color development, and browning of grape juice with time. Hence, the objectives of this study were: (1) to determine the degradation kinetics of individual and total anthocyanins contained in grape juice stored at different temperatures for up to 360 days, and (2) to evaluate the relation between anthocyanin content and the change in polymeric color, juice browning and haze development during storage.

## Materials and methods

### Chemicals and materials

The grape juice of both grape cultivars (“Ruby” and “Merlot”) was obtained from a local juice processor and stored at −35°C until use. Standards were purchased as follows: M3G from Chromatographic Specialities Inc. (Brockville, ON, Canada), Pt3G, Pn3G, and D3G from Polyphenols Laboratories (Sandnes, NOR) and C3G from Extrasynthese (Genay Cedex, FRN). Potassium chloride, hydrochloric acid, sodium acetate, methanol, acetonitrile, and phosphoric acid were purchased from Sigma Aldrich (Oakville, ON, Canada).

### Sample preparation

An aliquot of 100 mL juice was filled into amber bottles and pasteurized in a water bath at 70°C for 15 min. The headspace was flushed with nitrogen gas closed tightly using screw caps equipped with rubber septa, first sealed with Parafilm M (Bemis Flexible Packaging, Neenha, WI) film and stored at 5, 25, 35°C. Two bottles of juice samples were removed at 0, 5, 10, 20, 30, 60, 120, 210, 280 and 360 days and kept at −80°C until analyses. Extraction and purification of anthocyanin pigments were performed using 100% methanol and C_18_ column by solid phase extraction protocol (SPE; Strata™-X 25 μm 200 mg/3 mL) according to the manufacturer's recommendation (Phenomenex, Torrance, CA, USA).

### Determination of total anthocyanins and polymeric color

The total monomeric anthocyanin content and polymeric color were determined by pH differential and bisulfite bleaching methods, respectively (21, 22). A UV-visible spectrophotometer (Perkin-Elmer Lambda 20, Waltham, MA, USA) and 1 cm path length disposable cuvettes were used to measure absorbance at 420, 520, and 700 nm respectively. The total anthocyanin content was expressed as M3G using an extinction coefficient of 28,000 L/cm.mg and molecular weight of 493.43 g/mol. All the measurements were duplicated.

### Quantification of selected anthocyanins

Analyses of selected individual anthocyanins were performed according to a previously described method (23) using LC-MS system (Alliance 2,695 separations module, Waters, Milford, MA, USA) equipped with a photodiode array detector (Waters 2998) and Masslynx V4.0 data analysis system (Micromass, Cary, NC, USA). Briefly, the separation was carried out by a C_18_ Phenomenex (Torrance, CA, USA) Luna column (150 × 2.1 mm, 5 μm) maintained at 25°C. The flow rate was 0.35 mL/min with a total run time of 42 min and an injection volume of 20 mL. The eluents were 5% (vol/vol) formic acid in water (A) and 5% (vol/vol) formic acid in methanol (B). A linear gradient profile was used with the following proportion of solvent A applied at time *t* (min): (*t*, A%): (0, 90%), (10, 70%), (17, 60%), (21, 48.8%), (26, 36%), (30, 10%), (31, 90%), (37, 90%). The standards were prepared in methanol, and their concentration was as follows 0.25-25 mg/L C3G, M3G, D3G, Pt3G, and Pn3G. Each analysis was carried out in duplicate.

### Determination of browning index

Browning index (BI) which shows the ratio of the loss of total anthocyanin pigment to the development of brown color was calculated using Equation 1. Absorbance was measured after diluting the juice with distilled water (1:1).

(1) $$\begin{array}{r} \text{BI }=\;\left(\text{A }420\text{ nm}\right)/\left(\text{A }520\text{ nm}\right) \end{array}$$

### Haze measurement

Turbidity measurements were carried out using a turbidimeter (Hach 2100AN, London, ON, Canada). Undiluted juice samples were poured in a 10 mL cell holder, which was cleaned and air-dried, and haze sediments were re-suspended by gently rocking the sample holder just prior to measurement. All samples were allowed to equilibrate to room temperature before measuring their turbidity. Results were expressed in nephelos turbidity units (NTU).

### Degradation kinetics

Instead of the commonly used first-order model (Equation 2), which assumes the complete degradation of anthocyanins after long-term storage, a first-order model which assumes an equilibrium value, *Ae* (Equation 3) first devised by (24) was applied. This model has been used in grape (25) and strawberry juices (26, 27) to express the anthocyanin degradation after ozonation and sonication processes.

(2) $$\begin{array}{rcl} A_{t} & = & {\text{ A}}_{o}*\text{exp}\left(-kt\right) \end{array}$$

(3) $$\begin{array}{rcl} A_{\text{t}} & = & \;\left(A_{\text{o}}-A_{\text{e}}\right)*exp\left(-kt\right)\;+\;A_{\text{e}} \end{array}$$

where *A*_*o*_ is the initial anthocyanin content, *A*_*e*_ is the equilibrium value, *A*_*t*_ is the anthocyanin content after treatment time *t* (min) and *k* is the rate constant (day^−1^).

The half-life (*t*_1/2_) (the time needed for 50% degradation of anthocyanins) and activation energy (E_a_), a measure of temperature dependence, were calculated using the Equations (4, 5), respectively.

(4) $$\begin{array}{rcl} t{\;}_{1/2} & = & \text{Ln }2/k \end{array}$$

(5) $$\begin{array}{rcl} \text{Log }k_{t}/k_{o} & = & {\text{E}}_{\text{a}}/\left(2.303\text{R}\right)\left[1/\text{T}1-1/\text{T}2\right] \end{array}$$

where *k* is rate constant (day^−1^), *k*_*o*_ is the frequency factor (day^−1^), R is gas constant (8.314 J/mol.K) and T1 and T2 are absolute temperatures in Kelvin (K).

### Statistical analysis

The experimental design consisted of a three-factor (3 × 2 × 10; Temperature; 5, 25, and 35°C, Cultivar; “Merlot” & “Ruby” and Storage duration; 0, 5, 10, 20, 30, 60, 120, 210, 280, and 360 days) storage trial with two replications. Nonlinear regression analysis was performed using SYSTAT 11 (San Jose, CA, USA) and Minitab 16 (Coventry, United Kingdom) statistical software applications. Analysis of variance (ANOVA) was applied to compare the initial concentration of individual anthocyanins between the two cultivars. The possibility of significant (*p* < 0.05) differences between means was evaluated using Tukey's test.

## Results and discussion

### Changes in anthocyanin concentration

The main anthocyanin pigments found in “Ruby” and “Merlot” grape juice were analyzed before and during storage at 5, 25, and 35°C. The selected two grape cultivars represent commercially grown common grape cultivars in Eastern Canada. The three selected temperatures represented refrigerated, room temperature and abused storage/transport temperature conditions. In line with other studies (23, 28, 29) five major individual anthocyanins namely M3G (45.20–47.95 mg/L), D3G (22.0–25.15 mg/L), Pt3G (17.35–19.35 mg/L), Pn3G, (12.89–13.49 mg/L), and C3G (2.49–3.847 mg/L) were identified from “Ruby” and “Merlot” grape juices, respectively (Table 1). In agreement with previous studies (23, 28, 29), our results found that M3G as the predominant anthocyanin compound in both grape cultivars and accounted for 43.6–45.6% of the total anthocyanins (Table 1). Our findings also indicated that grape juice from “Merlot” had significantly higher (*p* < 0.05) content of all individual anthocyanins as compared to “Ruby.” The differences in anthocyanin concentrations are presumably due to genetic variation between the two cultivars, is in accordance with a study conducted by Liang et al. (28).

**Table 1:** Initial anthocyanin concentration in grape juices from “Merlot” and “Ruby” cultivars.

**Concentration of anthocyanin compounds (mg/L)**^**a**^					
	**M3G^b^**	**D3G**	**Pt3G**	**Pn3G**	**C3G**
**CULTIVAR**					
“Merlot”	47.95 ± 0.49a	25.15 ± 1.02a	19.35 ± 0.15a	13.49 ± 0.34a	3.85 ± 0.01a
“Ruby”	45.20 ± 0.73b	22.01 ± 0.50b	17.35 ± 0.15b	12.89 ± 0.04b	2.49 ± 0.01b

a *Values are expressed as mean ± SE (n = 2)*.

b *C3G, cyanidin-3-O-glucoside; Pt3G, petunidin-3-O-glucoside; D3G, delphinidin-3-O-glucoside, Pn3G, peonidin-3-O-glucoside and M3G, malvidin-3-O-glucoside*.

### Degradation kinetics of anthocyanins

**Figure 1:**
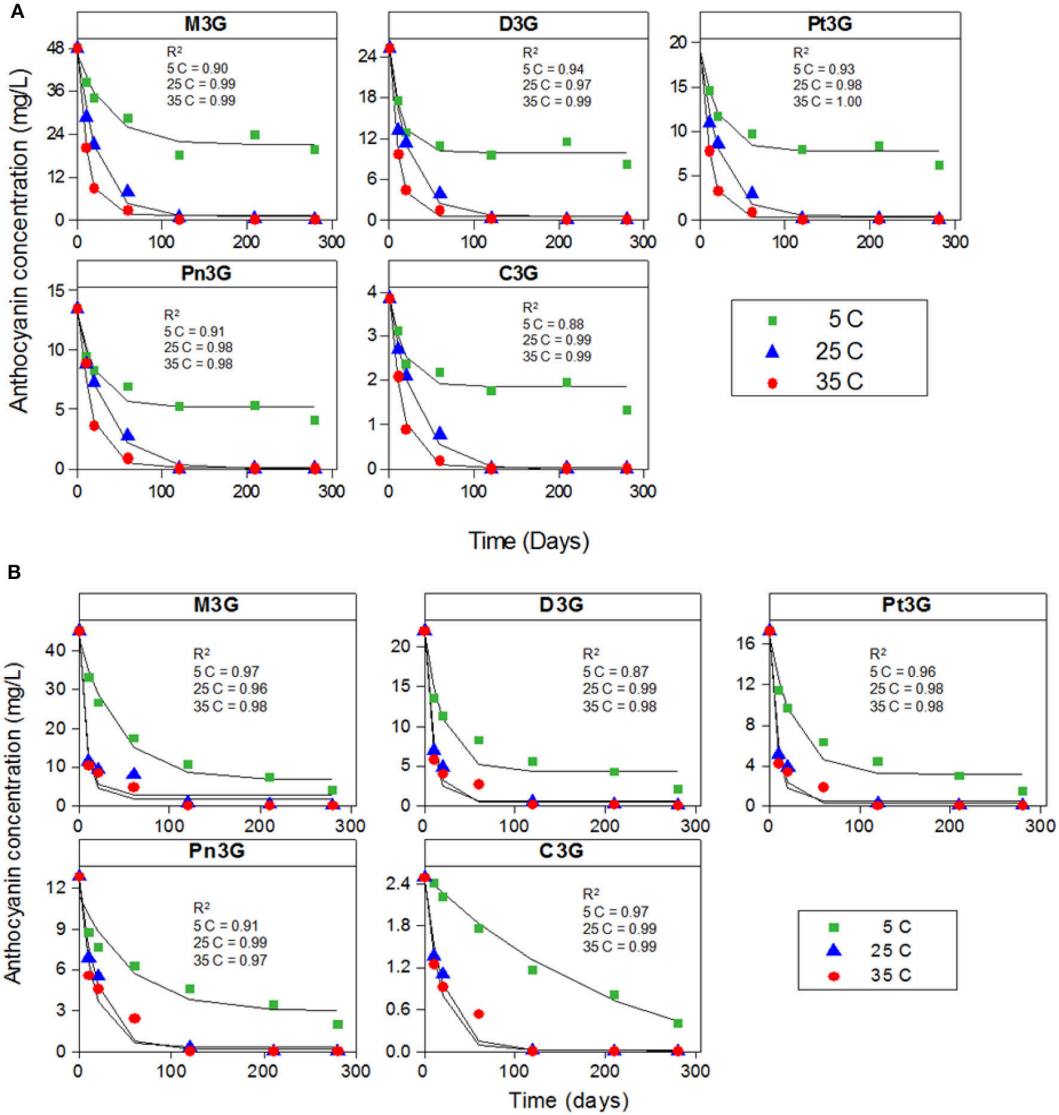
The first-order degradation kinetics plots of individual anthocyanins in juices from “Merlot” **(A)** and “Ruby” **(B)** grape cultivars during storage at (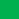) 5, (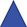) 25, and (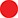) 35°C. C3G, cyanidin-3-O-glucoside; Pt3G, petunidin-3-O-glucoside; D3G, delphinidin-3-O-glucoside; Pn3G, peonidin-3-O-glucoside; M3G, malvidin-3-O-glucoside.

**Figure 2:**
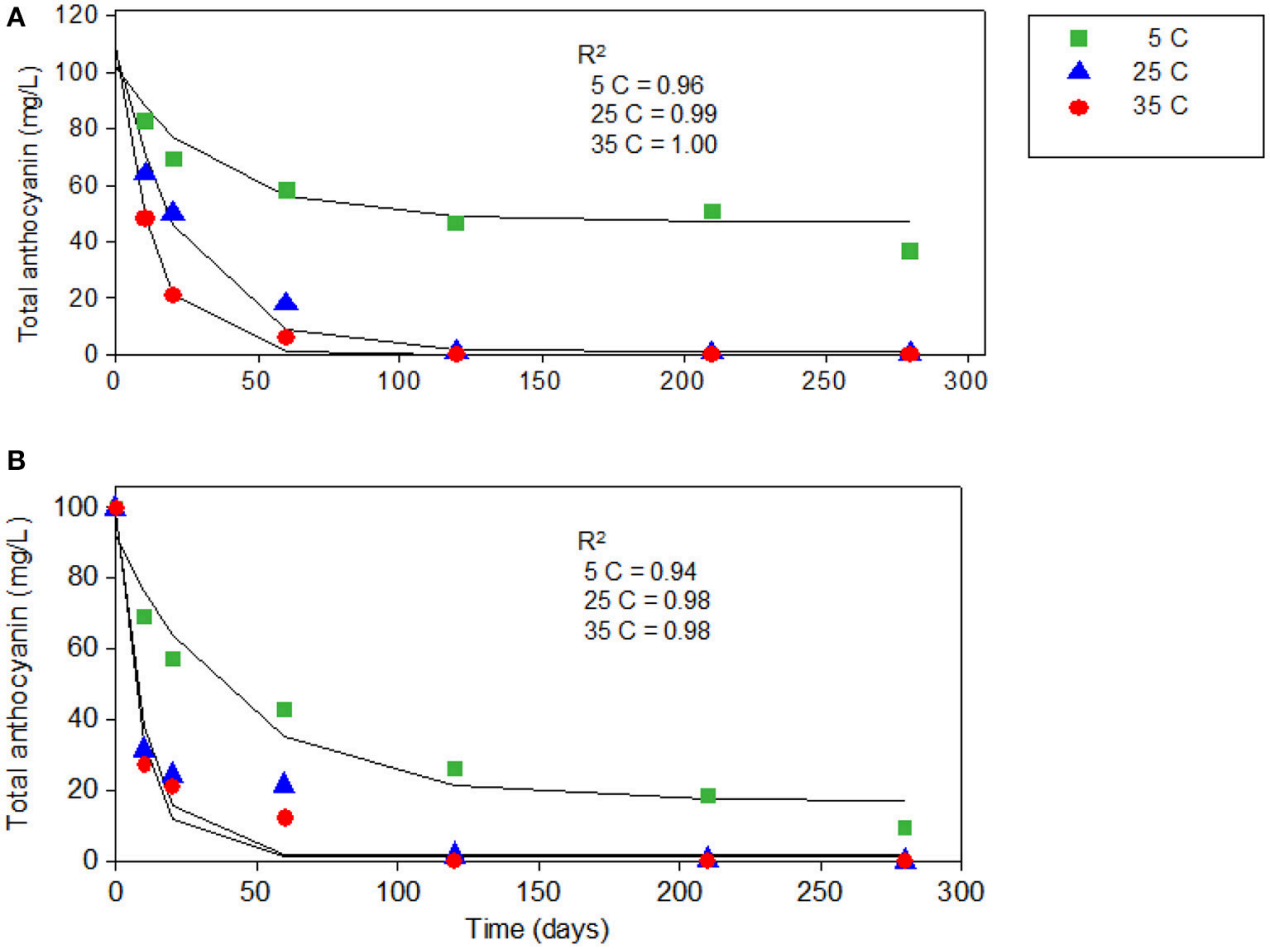
The first-order degradation kinetics plots of total anthocyanins measured by HPLC in juices from “Merlot” **(A)** and “Ruby” **(B)** grape cultivars stored at (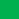) 5, (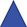) 25, and (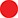) 35°C.

The degradation kinetics of individual and total anthocyanins was determined at different storage temperatures (5, 25, and 35°C) up to 280 days of storage (Figures 1, 2). The corresponding kinetic parameters (*k, t*_1/2_, _and_ E_a_) were indicated in Table 2. Irrespective of cultivar, the decay of all the individual anthocyanins as a function of storage time at 5, 25, and 35°C were fitted well to the first-order model containing an equilibrium term (Equation 3) (*p* < 0.05) (Figure 1). For all individual anthocyanins, the coefficients of determination (*R*^2^) were > 0.96 especially at 25 and 35°C, demonstrating a direct correlation between anthocyanin concentration decrease and storage time at each temperature (Figure 1). A similar degradation model was reported for C3G in ozonated strawberry and grape juices (25, 26). While no complete loss of anthocyanins was noted, other studies on blackberry juice (30), strawberry juice (31), sour cherry juice (32), and pomegranate juice (14) reported first-order degradation of individual or total anthocyanins according to (Equation 2).

**Table 2:** Degradation kinetic parameters of anthocyanins of “Merlot” and “Ruby” grape juices stored at 5, 25, and 35°C.

**Compounds^a^**	**Cultivar**	**Temp.(^°^C)**	***k* (days^−1^)**	***R*^2^**	***A_*o*_* (mg/L)**	***A_*e*_* (mg/L)**	***t_1/2_*(days)**	**E_a_ (kJ/mol)**	***r*^2^**
M3G	“Merlot”	5	0.03	0.90	46.17	20.92	25.67	26.57	0.94
		25	0.04	0.99	46.67	0.97	16.50	
		35	0.09	0.99	47.92	1.28	7.62	
	“Ruby”	5	0.02	0.97	42.83	6.58	34.66	49.63	0.91
		25	0.14	0.96	44.94	2.70	4.95	
		35	0.14	0.98	44.95	1.77	4.95	
Pt3G	“Merlot”	5	0.04	0.93	19.13	7.74	17.33	17.33	0.79
		25	0.05	0.98	18.73	0.31	13.86	
		35	0.09	1.00	19.33	0.19	7.70	
	“Ruby”	5	0.04	0.97	16.96	3.89	15.75	26.52	0.98
		25	0.11	0.98	17.17	0.29	6.48	
		35	0.13	0.98	17.22	0.46	5.33	
D3G	“Merlot”	5	0.04	0.83	25.22	9.88	17.33	18.42	0.79
		25	0.05	0.97	24.40	0.39	13.86	
		35	0.10	0.99	25.09	0.38	7.29	
	“Ruby”	5	0.01	0.87	21.42	4.28	69.31	63.30	0.94
		25	0.10	0.99	21.80	0.43	6.73	
		35	0.12	0.98	21.87	0.63	5.55	
Pn3G	“Merlot”	5	0.03	0.91	13.09	5.17	23.10	11.75	0.79
		25	0.04	0.98	13.02	0.00	17.33	
		35	0.05	0.98	13.73	0.04	13.86	
	“Ruby”	5	0.02	0.91	11.45	2.94	36.48	29.75	0.99
		25	0.05	0.99	12.63	0.11	25.67	
		35	0.07	0.97	12.51	0.27	16.50	
C3G	“Merlot”	5	0.03	0.88	3.83	1.74	23.10	19.69	0.99
		25	0.05	0.99	3.77	0.02	13.86	
		35	0.07	0.99	3.87	0.02	9.90	
	“Ruby”	5	0.00	0.97	2.51	0.47	173.29	66.5	0.94
		25	0.05	0.99	2.44	0.01	14.75	
		35	0.06	0.99	2.45	0.01	12.16	
Total ACY^b^ (HPLC)	“Merlot”	5	0.01	0.80	167.00	70.00	138.63	21.93	0.99
		25	0.01	0.97	188.94	6.70	99.02	
		35	0.01	0.99	178.00	9.90	86.64	
	“Ruby”	5	0.00	0.90	184.40	91.20	231.05	39.60	0.91
		25	0.00	0.96	173.89	40.01	173.29	
		35	0.01	0.99	181.00	14.00	115.52	
Total ACY^c^	“Merlot”	5	0.03	0.92	102.76	0.03	138.63	16.92	0.86
		25	0.04	0.99	107.52	0.04	99.02	
		35	0.08	1.00	109.82	0.08	86.64	
	“Ruby”	5	0.02	0.94	92.00	0.02	231.05	43.39	0.97
		25	0.09	0.98	98.00	0.09	173.29	
		35	0.12	0.98	98.00	0.12	115.52	

a *C3G, cyanidin-3-O-glucoside; Pt3G, petunidin-3-O-glucoside; D3G, delphinidin-3-O-glucoside; Pn3G, peonidin-3-O-glucoside and M3G, malvidin-3-O-glucoside*.

b *Total ACY (HPLC), the sum of the five major anthocyanin as quantified by HPLC*.

c *Total ACY, total anthocyanin by pH differential method expressed as M3G*.

In both cultivars, the individual anthocyanin content decreased considerably at the rate strongly dependent on storage temperature (Figure 1).The rate of decay was noticeably lower at 5°C (*k* = 0.01–0.04) as compared to higher storage temperatures 25°C (*k* = 0.04–0.14) and 35°C (*k* = 0.05–0.14) (Table 2). After 280 days of storage, juices stored at 25 and 35°C had dramatically higher loss of anthocyanins (95–99.9%) as compared to those stored at 5°C (74–81% in “Ruby” and 50–60% in “Merlot”) (Table 2). The immense loss of individual anthocyanins (70–91%) at higher temperatures was also reported in pomegranate juices stored at 20 and 37°C for 210 days (14).

The degradation kinetics of total anthocyanins had the same trend as observed in individual anthocyanins (Figure 2). After 280 days of storage, the total anthocyanin content of “Merlot” juice was reduced by 40% at 5°C, 86% at 25°C and almost 100% at 35°C (Table 2). Similarly, the total anthocyanin in a juice from “Ruby” had reduced by 70, 88, and 98% at 5, 25, and 35°C respectively (Table 2). In agreement with our results, a study conducted on blueberry juice reported more than 50% total anthocyanin loss after 6 months of storage (12).

Each anthocyanin compound had a different degradation rate and temperature sensitivity. In our study, the most temperature sensitive anthocyanin compounds were found in “Ruby” juice samples. These include C3G (Ea = 66.5 kJ/mol) and D3G (Ea = 63.3 kJ/mol) (Table 2). Similarly, large differences in the *t*_1/2_ values existed among the individual anthocyanins stored at the same or different temperatures in both cultivars (Table 2). Generally, our results indicated that in both cultivars, all anthocyanin compounds exhibited longer *t*_1/2_ values at 5°C as compared to 25 and 35°C storage temperatures, which is clearly observed in “Ruby” cultivars (Table 2). Of the individual anthocyanins, the highest *t*_1/2_ values were observed in C3G (*t*_1/2_ = 73 days) and D3G (*t*_1/2_ = 69 days) at 5°C (Table 2). However, at 25 and 35°C the same anthocyanin compounds showed a dramatic drop in their *t*_1/2_ values (Table 2). These results are in accordance with a study conducted on blueberry juice (33) where higher temperatures caused a faster degradation of D3G and C3G. The difference in stability among individual anthocyanins could be ascribed to their chemical structure. The D3G derivatives have three orthophenolic groups in the B ring and the C3G derivatives, which have the second order reactivity, have two orthophenolic groups (33). On the other hand, Pn3G, which has the least temperature sensitivity (Ea = 11.75 kJ/mol, “Merlot,” and 29.75 kJ/mol, “Ruby”) possesses only one phenolic substituent in the B ring. The accelerated degradation of anthocyanins at the higher temperature is also ascribed to the hydrolysis of the glycosidic bonds, which connect aglycones with glycosyl moieties (29). Aglycones (anthocyanidins), the basic structural skeleton (C6-C3-C6) of anthocyanins, are much less stable than their glycosylated counterparts are and hence the loss of sugars through hydrolysis of the glycosidic bonds will lead to faster degradation of anthocyanins (29). Generally, our results indicated that storing grape juice at 5°C could effectively reduce the loss of individual and total anthocyanins, especially from “Merlot” juice samples.

### Change in polymeric color, haze formation, and browning

**Figure 3:**
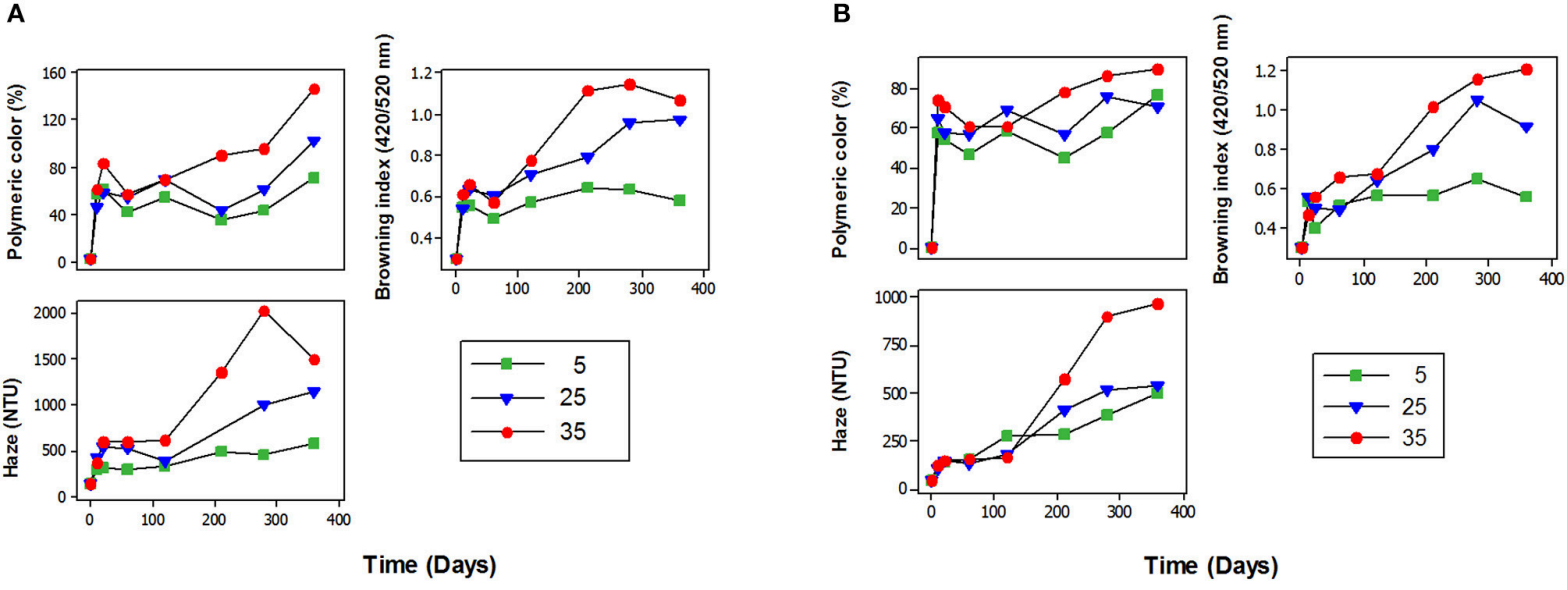
Polymeric color development, brown color development, and haze formation in juices from “Merlot” **(A)** and “Ruby” **(B)** grape cultivars stored at (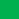) 5, (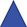) 25, and (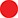) 35°C.

The development of polymeric color, haze formation, and browning of juice samples was also monitored up to 360 days of storage and presented in Figure 3. In both cultivars, higher temperature and longer storage periods caused not only reduced anthocyanin concentrations but also other changes including polymeric color accumulation, brown color development, and haze formation. The correlation between anthocyanin degradation and these physiochemical parameters is presented in Table 3.

**Table 3:** Correlation between total anthocyanin content, with browning index (A _420__nm_/A _520__nm_), polymeric color (%) and haze (NTU).

**Cultivar**	**Color and appearance**	**Total anthocyanin (mg/L) at different storage Temps (**^**°**^**C)**^**a**^		
		**5^°^C**	**25^°^C**	**35^°^C**
“Merlot”	Browning index	−0.72(0.023)^a^	−0.84(0.001)	−0.96(0.000)
	Polymeric color	−0.49(0.070)	−0.63(0.012)	−0.99(0.000)
	Haze	−0.77(0.001)	−0.69(0.015)	−0.898(0.001)
“Ruby”	Browning index	0.70(0.025)	0.98(0.000)	0.99(0.000)
	Polymeric color	−0.19(0.081)	−0.80(0.001)	−0.98(0.000)
	Haze	−0.84(0.001)	−0.34(0.061)	−0.57(0.070)

a *Values in parenthesis are p values*.

Polymeric color is a measure of pigmented polymers, which are resistant to SO_2_ bleaching (34). In both cultivars, polymeric color content increased considerably during storage at the rate clearly dependant on storage temperature (Figure 3). After 360 days of storage, juices stored at 25°C (76%, “Ruby”; 101%, “Merlot”) and 35°C (90%, “Ruby”; 95%, “Merlot”) had the higher polymeric color as compared to those stored at 5°C (70% “Merlot”; 71% “Ruby”) (Figure 3). Similarly other studies reported increased polymeric color in strawberry syrups (by 27%) stored for 60 days at room temperature (35), black carrot juice concentrate (by 31%) stored for 319 days at 30°C (36) and blueberry juice (by 15%) stored for 6-months at 25°C (12). The increasing trend of polymeric color during storage could indicate the extensive polymerization of anthocyanins during storage (37) as our results showed a highly significant (*p* < 0.001) negative correlation (*r*^2^ = 0.96, “Ruby”; *r*^2^ = 0.99, “Merlot”) between polymeric color and total anthocyanin content at 35°C (Table 3). Hence, our results suggested that the loss of anthocyanins during storage was accompanied by the increased polymeric color values, especially at higher storage temperatures. Polymerisation reactions could be due to endogenous enzymes including residual peroxidase activity (10, 11) and residual polyphenol oxidase activity (38) that were not inactivated entirely by the pasteurization process. In addition, higher storage temperatures could trigger these reactions, since lower temperature slows enzymatic activity and may have been a rate-limiting step during storage (39). Another potential mechanism for polymerization could be condensation reactions of anthocyanins with other phenolic compounds such as tannins (12).

Browning of fruit juice is another aspect of quality deterioration, which is measured as BI in this study. In both cultivars, BI values increased considerably during storage at the rate clearly dependant on storage temperature (Figure 3). After 360 days of storage juice samples from both cultivars had considerably higher BI values at 25 (67–69%) and 35°C (71–75%) as compared to 5°C (46–48%) storage temperature (Figure 3). The increased BI at higher temperatures indicates the accumulation of brown color at the expense of reduced red anthocyanin pigments which was confirmed by the significant (*p* < 0.05) negative correlation between BI and total anthocyanin content at 5 (*r*^2^ = 0.72), 25 (*r*^2^ = 0.84) and 35°C (*r*^2^ = 0.96) (Table 3). This result is consistent with other studies which reported increased BI in black carrot juice stored at 40°C for 90 days (40) and apple juice stored at 37 and 25°C (41) for 180 days. In fruit juices, browning is associated with enzymatic or non-enzymatic Maillard browning reactions (9). However, in our experiment, since the juice samples were pasteurized and bottled under nitrogen gas with the exclusion of oxygen, it is suggested that the non-enzymatic Maillard browning which is due to the reaction between reducing sugars with free amino acids, could be the main reason for the brown color development during storage. However, enzymatic browning due to oxidation of polyphenols by the residual polyphenol oxidase enzymes could also have some contribution (42, 43).

Haze formation can limit the shelf life of products that the consumer expects to be clear including fruit juices. Haze also fouls process equipment surfaces with deposits that are difficult to remove by in-place cleaning (44). Irrespective of cultivar, our results found a substantial increase in haze values at 25 (86–90%) and 35°C (93–94%) as compared to 5°C (69–87%) during storage up to 360 days (Figure 3). Our results also indicated a significant negative (*p* < 0.05) correlation between haze development and anthocyanins concentration particularly in “Merlot” juices. Moreover, in agreement with (45), haze showed a pattern of change similar to polymeric and brown color development and opposite to anthocyanin pigment concentration. Hence, it can be suggested that the haze development could be linked with anthocyanin polymerization and brown color formation. The most reported cause of haze in beer, wine, and fruit juices is the protein-polyphenol interactions that grow to colloidal size and become insoluble, scatter light and result in turbidity, which may be visible as a haze (46–48). Another study indicated a relation between enzymatic browning and haze formation (49). During enzymatic browning, the polyphenol oxidase acts on phenolic substances, which may result in the formation of condensed tannins that are the precursor of haze by interacting with proteins. This means enzymatic browning might accelerate haze formation (49). The increased haze formation at higher temperature might be ascribed to the higher molecular mobility and/or reactivity of haze active molecules at 25 and 35°C compared to 5°C, thus allowing more interactions to take place between the compounds (41).

As presented in Figure 3, the trend of increased haze development from both cultivars indicates four phases including the initial growth phase, the lag phase, the second growth phase, and termination phase. The haze development trend observed in our study is different from previous studies in packaged beer (50) and apple juice (41) which have shown only three phases (lag, growth, and termination) but not the initial growth phase. The initial growth phase observed during the first 10 days at all temperatures might be due to the remarkable increment of polymeric color and BI during the same period (Figure 3). However, since haze development is a complex multifactorial phenomenon (51) a number of different reactions might be involved. Among the three storage temperatures studied, the terminal phase was clearly visible for samples stored at 25 and 35°C. Haze development in juice samples stored at 5°C appeared to remain in the growth phase at the end of the storage period (Figure 3). The lag phase might be attributed to the formation of soluble protein-polyphenol complexes, which remained in solution as a stable sub-colloidal particles and did not contribute to turbidity (47). The compounds involved in the lag phase may not be all haze-active and should undergo the required reaction of catalytic activity, such as oxidation or polymerization of the polyphenols to make them haze active (9, 52). Once haze-active compounds are formed, interactions among these active haze precursors result in rapid formation of haze during the second phase (the growth phase). These rapid reactions resulted in a greater increase in turbidity values as depicted in the growth phase. Haze development lastly terminates (terminal phase) when there are no more active haze-precursors or binding sites for cross-linking (47, 48, 53).

## Conclusions

The results of this research provided evidence on the degradation kinetics of individual as well as total anthocyanin pigments contained in grape juice when stored at different temperatures for long periods of time. The five major individual anthocyanins identified from “Merlot” and “Ruby” grape juices were M3G, D3G, Pt3G, Pn3G, and C3G. During the long-term storage, total, and individual anthocyanins degraded following first-order reaction kinetics at the rate strongly dependent on temperature. The individual anthocyanins exhibited different degradation rates and kinetic parameters, which might be related to their chemical structure. At higher storage temperatures D3G and C3G were found the most unstable and temperature sensitive anthocyanins. Higher temperatures and longer storage periods not only reduced anthocyanin concentrations but also increased polymeric color accumulation, brown color development, and haze formation. Therefore, the findings could be adapted to industrial applications for preserving the biologically active anthocyanins and authentic color of grape juice by storing them at low temperatures for limited storage periods.

## Author contributions

BM conducted all the experiments, data analyzing and writing the first draft of the manuscript. HPVR and RAS contributed by advising methods and experimental design, supervising data analysis, and reviewing the manuscript.

### Conflict of interest statement

The authors declare that the research was conducted in the absence of any commercial or financial relationships that could be construed as a potential conflict of interest.
